# High throughput surface plasmon resonance imaging method for clinical detection of presence and strength of binding of IgM, IgG and IgA antibodies against SARS-CoV-2 during CoViD-19 infection

**DOI:** 10.1016/j.mex.2021.101432

**Published:** 2021-06-29

**Authors:** Richard B.M. Schasfoort, Jos van Weperen, Margot van Amsterdam, Judicaël Parisot, Jan Hendriks, Michelle Koerselman, Marcel Karperien, Anouk Mentink, Martin Bennink, Hans Krabbe, Leon WMM Terstappen, A.H. Leontine Mulder

**Affiliations:** a Department of Medical Cell BioPhysics, Faculty of Science and Technology, University of Twente, PO Box 217, 7500 AE Enschede, The Netherlands; b Vysens BV, PO Box 50035, 7550 LA Hengelo, The Netherlands; c Carterra, 825 N. 300 W., Suite C309, Salt Lake City, UT 84103, USA; d Department of Developmental BioEngineering, Faculty of Science and Technology, University of Twente, PO Box 217, 7500 AE Enschede, The Netherlands; e NanoBio research group, Saxion University of Applied Sciences, PO Box 70000, 7500 KB Enschede, The Netherlands; f Department of Clinical Chemistry and Laboratory Medicine, Medisch Spectrum Twente, PO Box 50000. 7500 KA Enschede, The Netherlands; g Department of Clinical Chemistry, Medlon BV, 7512 KZ Enschede, The Netherlands; h Department of Clinical Chemistry and Laboratory Medicine, Ziekenhuis Groep Twente, PO BOX 7600, 7600 SZ Almelo, The Netherlands

**Keywords:** Corona, Infectious diseases, Antibody isotypes, Affinity, Off-rate, High throughput, Biosensor

## Abstract

Surface Plasmon Resonance imaging (SPRi) was used to determine the presence and strength of binding of IgG, IgM and IgA against the Receptor Binding Domain (RBD) of SARS-CoV-2 in sera of 102 CoViD-19 and non-CoViD-19 patients. The SPRi assay simultaneously measures the antibody isotype levels and the strength of binding to the RBD of ultimate 384 patient samples in one run. It turns out that during the course of the disease, the IgG levels and strength of binding increased while generally the IgM and IgA levels go down. Recovered patients all show high strength of binding of the IgG type to the RBD protein. The anti-RBD immunoglobulins SPRi assay provides additional insights in the immune status of patients recovering from CoViD-19. This new high throughput method can be applied for the assessment of the quality of the immune reaction of healthy individuals to SARS-CoV-2 and its mutants in vaccination programs.

• Surface Plasmon Resonance imaging is an unprecedented technology for high throughput screening of antibody profiling of CoViD19 patients.
• Fingerprinting of isotypes IgM, IgG and IgA can be performed for 384 patients in one run.
• An affinity maturation effect was shown for patients recovering from CoViD19.

## Highlights

• Surface Plasmon Resonance imaging is an unprecedented technology for high throughput screening of antibody profiling of CoViD19 patients.
• Fingerprinting of isotypes IgM, IgG and IgA can be performed for 384 patients in one run.
• Severity of the disease correlates well with the total anti-RBD of SARS-CoV-2 concentration in CoViD19 patients
• Affinity equilibrium constant (K_D_) of the polyclonal antibody binding was directly proportional to the off-rate (k_d_) simplifying the screening.
• Screening of the strength of binding of anti RBD antibodies was possible in high throughput and in one run together with the isotype analysis in the LSA SPR imager.
• An affinity maturation effect was shown for patients recovering from CoViD19.
• A tool is now available to test the quality of the immune reaction of individuals to SARS-CoV-2 and its mutants in vaccination programs.

## Graphical abstract

**Figure:**
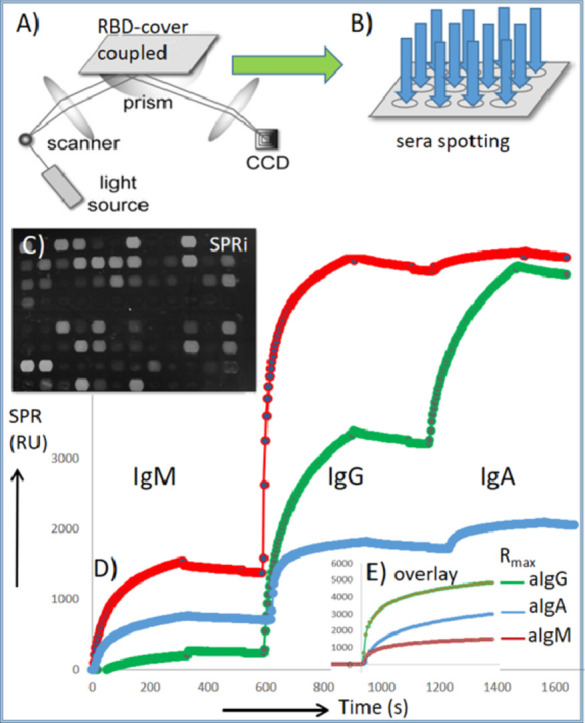

Specifications table

**Table:** 

Subject area:	Immunology and Microbiology
More specific subject area:	CoViD-19 and SARS-CoV-2, serology
Method name:	Clinical antibody detection generated by SARS-CoV-2 with high throughput Surface Plasmon Resonance imaging
Name and reference of original method:	Schasfoort, R.B.M., et al., 2021. Presence and strength of binding of IgM, IgG and IgA antibodies against SARS-CoV-2 during CoViD-19 infection. Biosensors and Bioelectronics [BIOS_113165] 183 (2021) https://doi.org/10.1016/j.bios.2021.113165
Resource availability:	BIOS 113165 Article reference: BIOS_BIOSBE-D-21-00090 Article title: Presence and strength of binding of IgM, IgG and IgA antibodies against SARS-CoV-2 during CoViD-19 infection Published in: Biosensors and Bioelectronics 183 (2021) https://doi.org/10.1016/j.bios.2021.113165 (original main article)

## Introduction

As indicated in the original condensed manuscript [Schasfoort, R.B.M., et al., 2021], a combined measurement of the anti-RBD IgM, IgG and IgA specific levels of CoViD-19 patients is possible in a single experiment using Surface Plasmon Resonance imaging (SPRi). In contrast, an ELISA immune assay need three separate tests to obtain these values for CoViD-19 patients but strength of RBD binding cannot be tested easily. The strength of binding of the anti-SARS-CoV-2 antibodies can be extracted from the SPRi data, while the levels of the antibodies are measured in high throughput for ultimate 384 patients simultaneously. This MethodsX article provides additional information on the materials and methods used that allow for the repetition of the experiments by other users of SPR instrumentation. Techniques, tricks and less successful results are also given, including 11 supporting figures for clarifying this new method.

## Material and methods

### Patient and control serum samples

Residual serum samples (*n* = 70) were obtained from 53 unique CoViD-19 patients confirmed by RT-qPCR and CT-scans and collected from March till June 2020. In total 20 out of 70 serum samples from cases were collected within 10 days after first symptoms (range 4–9 days), 50 were collected 10 or more days after first symptoms (range 10–28 days). From 10 patients more than 1 serum sample obtained at different time points was included.

Control non-SARS-CoV-2 samples (*n* = 49) were obtained from anonymous stored residual serum samples from healthy pregnant women collected in March 2019 (*n* = 37) and from 12 hospitalized patients with repetitive negative RT-PCR and a non-COVID explanation for their clinical symptoms.

The collected whole blood (3 ml) was allowed to clot by leaving it undisturbed at room temperature for 30 min. Then the sample was centrifuged at 2,500 x g for 5  min. The resulting supernatant is the designated serum and stored at -20° C. After thawing the serum, a 1:100 diluted sample with SPRi running buffer was prepared and pipetted in a microtiterplate for spotting on the RBD coated HC30M sensor surface using the Continuous Flow Microspotter (CFM, Wasatch Microfluidics, SLC, UT, USA).

Disease severity of the SARS-CoV2 infection was classified according to the WHO criteria [1] as either mild, moderate, severe or critical. Mild patients did not show abnormal CT imaging. Moderate patients had fever and/or classical respiratory symptoms, and typical CT images of viral pneumonia. Severe patients met at least one of the following additional conditions: (1) Shortness of breath with respiratory rate (RR) ≥30 times/min, (2) Oxygen saturation (SpO_2_, Resting state) ≤93%; or (3) PaO_2_/FiO_2_ ≤ 39.9 kPa. Critically ill patients met at least one of the extra following conditions: (1) Respiratory failure that required mechanical ventilation; (2) Shock; or (3) Multiple organ failure that required intensive care unit (ICU).

### ELISA

Euroimmun Anti-SARS-CoV-2 ELISA IgG assay (Euroimmun, Luebeck, Germany) was performed on serum samples on a Thunderbolt ELISA robot (Gold standard diagnostics, CA, US) according to the manufacturer's instructions. This ELISA provides a semi-quantitative in vitro determination of human IgG against SARS-CoV-2. The microplate wells are precoated with recombinant S1 structural protein. The results are evaluated by calculation of a ratio of the extinction of samples over the extinction of the calibrator. The sensitivity and specificity of samples > 10 days of disease duration are 80% and 99% respectively, according to the manufacturer (manual, March 2020).

The anti-SARS-CoV-2 Ig electrochemiluminescence (ECLIA) test (Roche Diagnostics, Rotkreutz, Switzerland), performed on the Cobas e 801 platform, detects antibodies to the recombinant nucleocapsid protein of SARS-CoV-2. The results are evaluated semi-quantitatively by calculation of the chemiluminescence of samples over the extinction of the calibrator. The sensitivity and specificity of samples > 14 days of disease duration according to the manufacturer are 99.5 % and 99.8 % respectively (manual, May 2020).

### Sensor preparation

For SPRi measurements the multiplex SPR imaging instrument (IBIS MX96, IBIS Technologies, Enschede, the Netherlands) and the Carterra LSA platform (Salt Lake City UT, US) were used with an installed sensor prism (HC30M, Xantec Bioanalytics Düsseldorf, Germany). Similar results for both instruments were obtained with this sensor surface. The sensor was prepared by first stabilization and removing the protective layer in water, followed by treatment with a 1:1 aqueous solution of 100 mM N-hydroxysuccinimide (NHS) and 400 mM N-ethyl-N’-(3-dimethylaminopropyl) carbodiimide hydrochloride (EDC) for 10 min. After rinsing with water for 20 s, the sensor was exposed for 20 min to the Spike RBD (40592-V08H, SINO Biological Frankfurt, Germany) in immobilization buffer (50 mM sodium acetate pH 4.8). Coupling with EDC-NHS yielded a reproducible sensor surface. After rinsing the sensor 20 s with water the surface was passivated with 1 M ethanolamine (pH 8.5) for 10 min. The sensor was then equilibrated in the running buffer composed of PBS (137 mM NaCl, 10 mM phosphate, 2.7 mM KCl, pH 7.4) supplemented with 1% bovine serum albumin (BSA) and 0.5% Casein and 0.1% Tween 20.

### Spotting sera

A Thunderbolt ELISA robot was used to dilute the serum samples in an optimized dilution ratio of 1:100. Each 2 µl serum was diluted with 198 µl of running buffer and pipetted in a 96 wells plate. For measurements on the IBIS MX96 a Continuous Flow Microfluidic (CFM) system (Carterra Salt Lake City UT, US) was used to capture 96 sera on a sensor functionalized with RBD-Spike. The first 48 samples were spotted in duplicate for 15 min. The Carterra LSA enables printing of 384 spots as 4 nested positions of 96 each. While the operation and injection of sample is similar for both instruments, the spotting process and dissociation rate can only be followed in real time on the LSA.

### Measurements on the IBIS MX96

The SUIT (Set Up Ibis Tool), DAX (Data acquisition software) and SPRINTX (Analysis software) software packages on the SPRI MX96 were used and Scrubber (BioLogic Software, Canberra, Australia) was used for the off-rate determination. After washing of the sensor chip spotted with patient sera, the sensor was first incubated with 50x diluted goat-anti-human-IgM (aIgM, 20-S5170 GND1-D0 Fitzgerald) in running buffer (200 µl for one run) and the second a 100x diluted goat-anti-human-IgG (aIgG-Fc, 20-S1211G001-S4 Fitzgerald) in SPRi running buffer. The third injection was with a 100x diluted goat-anti-human-IgA (aIgA, 20-S1111G000-S4 Fitzgerald). After converting the data by local referencing, zeroing the baseline and aligning the injection points of the three injections, the *R_max_* value was determined using a special biphasic fit algorithm (InterFluidics, Haaksbergen, The Netherlands). This software tool programmed using Microsoft ‘R’ Studio allows calculating the data on both SPR imagers. If the curve did not show an exponential behavior (e.g. negative samples) then a linear fit was applied and the average value of the linear fit was determined.

### Off-rate measurements on the LSA

In total 48 selected serum samples were spotted in duplicate in a single run on the HC30M RBD coupled sensor prism surface in 4 dilutions (1:50, 1:100, 1:200 and 1:400) to generate a 384-array. During the spotting process, the binding signals are followed for 15 min and each serum sample was measured 8 times at 4 dilutions. The signal recorded in RU mirrors the total anti-RBD antibodies bound. Following the spotting process, a 5 min injection of RBD (15 µg/ml) in dilution buffer resulted in sufficient dissociation of the anti-RBD antibodies. For all 384 spots, the global dissociation- or global off-rate constant was calculated. The final step consisted of sequential injections of solutions of anti-IgM, anti-IgG and anti-IgA antibodies. The ratio of bound immunoglobulins was calculated by determining the *R_max_* values from the anti-isotype antibodies binding signals. The *R_max_* value has a direct relation with the concentration of anti-RBD antibodies in serum.

### Sensor surface preparation, tricks and failure description

Initially a selection was made for which protein of SARS-CoV-2 the SPR imaging test should be set up. Among the various immunogenic proteins, we observed a better specific response on RBD with respect to S1 or S1+S2 or nucleocapsid proteins of SARS-CoV-2. The molecular weight of RBD with 192 amino acids [6] is smaller than the S domains and immobilization resulted in a higher functional ligand density. Based on these first results we decided to choose for the recombinant RBD-Spike protein and not to optimize the system for the various antigens. As confirmed in the literature, the RBD-Spike protein generates more potent antibodies for neutralizing the infection than the other immunogenic proteins of the virus [2,3]. In ELISA a discrimination between the isotypes is not possible in a single test, while in SPRi, the binding of anti-IgM (M), anti-IgG (G) and anti-IgA (A) can be followed in real-time after the injection of the specific antisera. For the sequence of the isotypes M, G and A, six permutations do exist: MGA, MAG, GMA, GAM, AMG and AGM. One can expect that there will be steric hindrance, which will influence the *R_max_* value. Be aware that detection of M is carried out with an anti-IgM of the IgG-type. Because the M showed the highest variation, we decided that M should be injected first. Because G is relevant for humoral immunity and is always tested in competing immunoassays G will be in the second injection so A was the third. All additional tests were carried out with the MGA sequence. Additionally the MGA sequence resulted in the lowest non-specific binding and/or false-positives.

During the process of developing the method, the blocking buffer is crucial for reducing non-specific binding. False-positives are due to a non-optimized blocking buffer. Although we definitely think that the blocking buffer can be improved further, we choose for PBS-Tween 20 (0.1%), BSA (0.5%) and casein (0.5%) in both the spotter and SPRimager.

The surface chemistry in terms of hydrogel quality was studied. Among various surface chemistries the HC30M surface of Xantec bioanalytics (Duesseldorf, Germany) turned out to be the best in terms of signal quality, signal homogeneity and signal reproducibility. This surface with a 30 nm open hydrogel was applied in both SPR-imagers.

XanTec Bioanalytics developed the first cross-platform, ready-to-use COVID-19 SPR sensor chip (code: C19RBDHC30M). It consists of SARS-CoV-2 RBD protein homogenously pre-immobilized on XanTec's proprietary ultra-low-background polycarboxylate HC surface. This new surface is a versatile tool for various applications in clinical and pharmaceutical COVID-19 R&D. Using the RBD protein in combination with the bioinert polycarboxylate surface ensures high specificity and sensitivity and also prevents false-positive results from samples from patients with earlier coronavirus infections or high nonspecific binding. With the COVID-19 RBD sensor chip and tailored reagents, XanTec brings considerable added value to pharmaceutical, vaccine and diagnostic research.

## Results

In this section, additional results are shown in 11 Figs. that supports the condensed article (Schasfoort, R.B.M. et al. 2021). It enables other researchers to repeat the SPR-experiments more easily using other detection technologies than the LSA of Carterra (SLC, UT, US) or IBIS MX96 (IBIS Technologies, Enschede, The Netherlands) but also other label free biomolecular interaction sensing instruments. In the figure captions, one can find details of features, benefits and tricks.

## Discussion

**Fig. 1:**
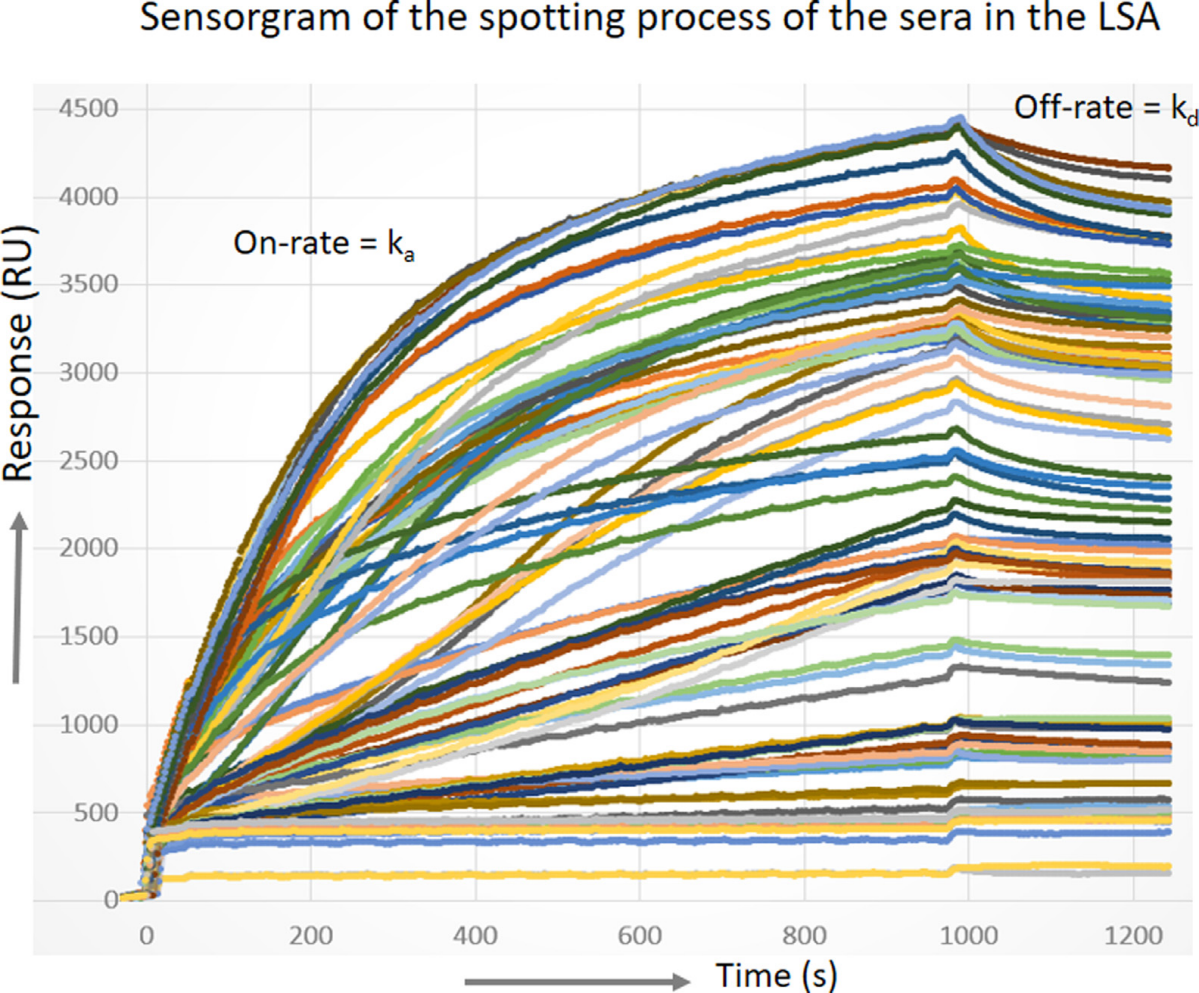
Raw non-referenced sensorgrams of the binding process of the CoViD-19 sera to the RBD coupled sensor surface using the LSA SPR imager. Because of mixed compositions of IgM, IgG and IgA and unknown concentrations in polyclonal mixture including non-specific binding, the association rate can not be determined by fitting of the binding curves. For the off-rate determination, it is necessary to obtain the referenced value of the SPR response in RU after the association phase and this is measured in the LSA (and not in the MX96). So, the end-value (RU) after printing the sera is applied to calculate the off-rate (k_d_) (see Fig. 2). Hence, the determination of an apparent concentration for each sample is not necessary and the calculation of k_d_ can be automated. In Fig. 11, a control experiment is shown that confirms a strong correlation of the affinity equilibrium dissociation constant (K_D_) with the off-rate (k_d_).

**Fig. 2:**
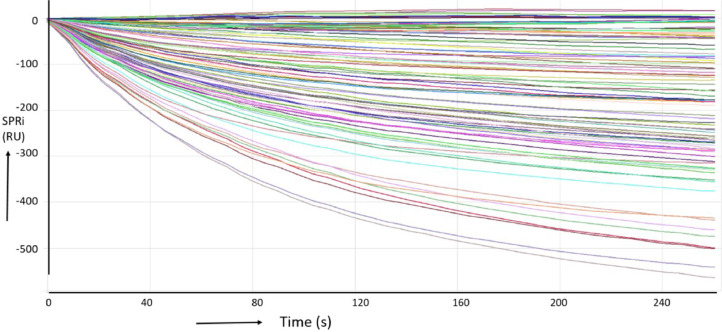
In this figure the dissociation phase is shown of immunoglobulins dissociating from the RBD coupled sensor surface by injecting free RBD protein (15 µg/ml) to prevent rebinding. In this graph, the end of the association phase is zeroed and aligned for calculating the slopes. As explained in the previous Fig. 1, the off-rate determination needs for each curve the response level RU after spotting the sera. The apparent off rate constant can be calculated accurately by the slope (RU/s) (Fig. 2) divided by the response (RU) (Fig. 1). In this way, the k_d_ (s^−1^) value for each curve can be calculated for all spotted sera in high-throughput. During the maturation of the immune response determined from longitudinal samples, the dissociation rate will decrease and the patients will generate better quality antibodies.

Both SPR imagers can be applied for detection of the composition and affinity of the serum isotype antibodies of CoViD19 patients.The specific anti-RBD antibody concentration cannot be determined easily because of the polyclonality of the serum that generates antibodies to all kinds of epitopes of SARS-CoV2. Only an averaged specific response by measuring the slope at the beginning of the association curve is a way to find an apparent concentration. But this specific response is a result of the anti RBD antibodies for all isotypes together. As can be seen in Fig. 1 the slopes, rates and definitely specific concentrations vary greatly. So, beforehand we don't know the specific anti-RBD IgM/IgG/IgA-concentration. The advantage of the LSA is that the spotting process of the sera can be followed in real-time and the off-rate can be measured in high throughput. However, in the IBIS MX96 the spot density can be calculated from the local ligand density after spotting the patient sera (see Fig. 4.), but this value is less accurate than tracking the real binding curves for all spots simultaneously. A better way of calculating the off-rate in the IBIS MX96 is by measuring the baseline drift (Fig. 2.) just after spotting divided by the total MGA response (Fig. 7). However, after installing the sensor in the MX96 the measurement should be started immediately because the drift of the baseline reflecting the off-rate of the serum antibodies on the sensor starts already after exposing the sensor to running buffer solution. A trick of controlled starting the dissociation process is by injecting an elution buffer containing e.g. RBD (at least 15 µg/ml or higher) in the IBIS MX96 or other component that minimizes the rebinding process of dissociating molecules. In this way, accurate ranking data of the off-rate values can also be obtained in the IBIS MX96.

During the development of the high throughput method, we tried an indirect route for the IBIS MX96 to reveal the strength of binding as shown in Fig. 10. Because the association rate could not be followed in the MX96 SPR imager during the spotting process, the off-rate can be accurately measured when the SPR response is known. The Fab anti human IgG injection (instead of the dimer anti-human IgG) is needed to determine the human IgG density on the spot and the Fab will not cross-link the human IgG's on the spot. The disadvantage of this indirect approach is that we cannot determine the fraction of the IgM and IgA off-rate. As Fig. 10 shows, we observed various off-rates of the anti-RBD antibodies of the IgG type but do not know the overall off-rate or strength of binding. So Fig. 10 is without the complex forming anti-isotype antibodies but with a Fab anti-human IgG. Hence, crosslinking to a precipitate will not occur and one can measure clearly the off-rate of this secondary binding event after the association phase with Fab anti-human IgG. We did not performed an experiment by injecting RBD in the dissociation phase because the method to determine the off-rate at *R* = 1000 means that the rebinding effect for all samples will be theoretically the same for this secondary interaction. And injecting RBD in the secondary dissociation phase complicates the secondary binding event because RBD injection induces higher off-rates in the primary phase. Then rebinding in the primary dissocation phase will not be similar. Because of this complicated way of finding off-rates, we only used the data from Fig. 1 with RBD in the dissociation phase. So the method of Fig. 10 is reliable and reproducible but not correct and complicated to measure. This is a message for readers of the manuscript that the preferred method is described in Fig. 1 and Fig. 2.

As explained in the paper of Schasfoort et al. [[4])], the off-rate is a function of the ligand density due to rebinding of the protein to the spots. When spots possess exactly the same ligand density then a comparison can be made for the apparent affinity equilibrium constant (K_D_) and/or the off-rate (k_d_). The four dilutions are needed to measure the off-rate by interpolating to R=1000 RU. In this way, the interpolated spot density at 1000 RU can be compared to each other for the sera and its serial dilutions. The absolute value of the off-rate depends on the quality of the Fab but it will be a constant for all spots if the antibodies will not dissociate from the surface. The quantitative value for all spots depends on the off-rates of the polyclonal human anti-RBD IgG and the samples can be compared/ranked to each other. This was the initial set-up to measure differences in off-rate in the IBIS MX96.

**Fig. 3:**
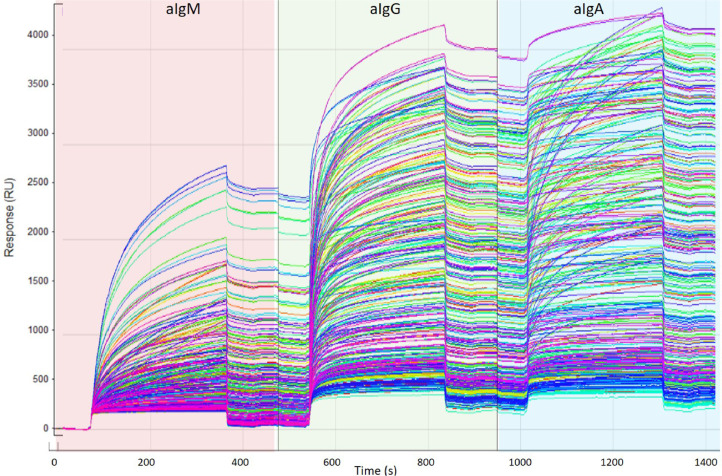
Raw non-referenced, non-zeroed sensorgrams of 384 spots after concatenated injections of anti-IgM, anti-IgG and anti-IgA antibodies using the LSA SPR imager. Clearly the various responses and the double duplicated readings of the 48 samples of the spotted sera can be observed.

Fortunately, in the LSA SPR imager, we can measure the binding of anti-RBD antibodies of all isotypes together directly from the patient sera in real time (see Fig. 1). So a Fab injection that shows an indirect off-rate is not necessary. This facilitates the assay enormously because a separate indirect test is not necessary. After the binding of the sera, we injected free RBD to prevent rebinding to the sensor and we could follow the dissociation process of the total anti-RBD antibodies much more accurate not only as a mixed total antibody dissociation. So, the value we calculate, is a measure of the strength of the complex bound to RBD. As can be observed in Fig. 2, the anti-RBD molecules will dissociate from the spots and definitely, this will affect the initial values of IgM, IgG and IgA. Now the remaining immunoglobulins are measured. We found that the difference between initial and remaining immunoglobulins was within the general error of the assay. Ultimately, the procedure was used to calculate the apparent off-rate (*k_d_*) of the anti-RBD antibodies accurately as is given in Fig. 3 of the original paper [5].

**Fig. 4:**
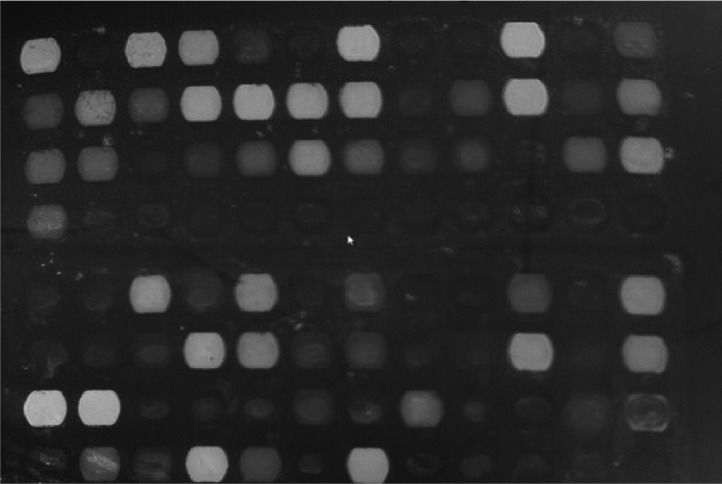
SPR reflection image of 96 COVID19 patients spotted on the sensor in a 8 × 12 array detected in the IBIS MX96 SPR imager. The bright spots are from patients with high titers. The generated sensorgrams in tile view are shown in Fig. 5.

**Fig. 5:**
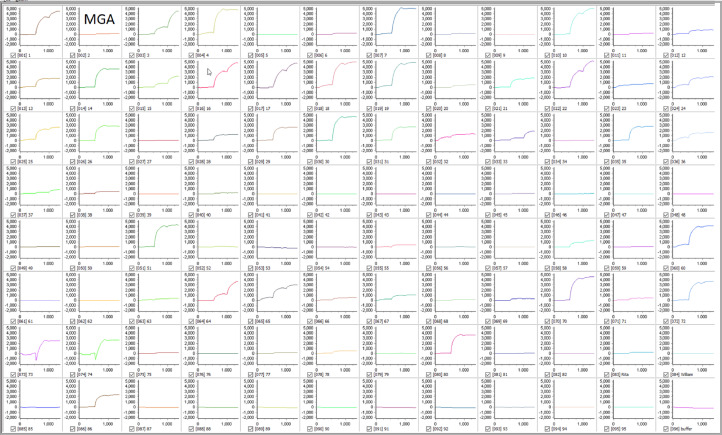
Tile view of interactions of anti-IgM, anti-IgG and anti-IgA sequence of injections on 96 serum spots using the IBIS MX96. All spots are exposed to the same composition of analyte but will respond differently because of patient dependent variation in presence of IgM IgG and IgA antibodies. The SPR reflection image is shown in fig. 4.

**Fig. 6:**
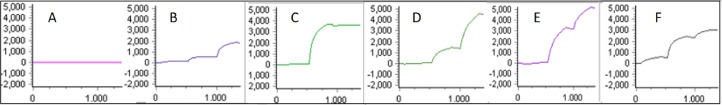
Six typical SPRi sensorgrams of anti-SARS-CoV-2 IgM, IgG and IgA in serum of CoViD-19 patients measured using the IBIS MX96. Panel **A**: patient without detectable immunoglobulins. **B**: patient with mild symptoms. **C**: patient with only an IgG response. **D**: patient with undetectable IgM a moderate IgG and a high IgA. Panel **E** and **F** show various levels of IgM, IgG and IgA.

**Fig. 7:**
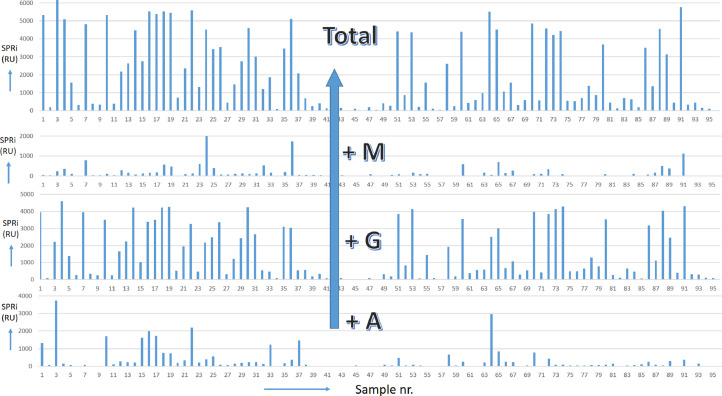
R_max_ values (RU) of sensorgrams from 96 spotted sera in a single high-throughput experiment after concatenated injections of anti-IgM, anti-IgG and anti-IgA. The total end value can be used to calculate the apparent off-rate for ranking the off-rates for all patients. (in combination with fig. 2)

**Fig. 8:**
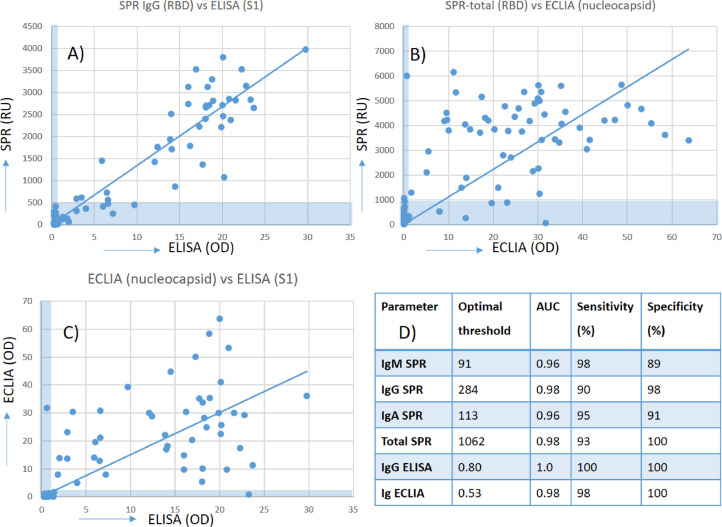
Correlation plot of the SPRi (Y-axis in RU), ELISA (optical density, X-axis OD) and ECLIA (X-axis OD) assays including the table of optimal threshold, AUC and sensitivity and specificity of the test. ECLIA based on the nucleocapsid protein (NP) shows less correlation with respect to the S1 (Elisa) and RBD (SPRi) assays. The immune response of patients with CoViD-19 varies enormously with respect to the RBD and NP antigens. The levels of threshold are drawn in the graphs. The type of antigen determines the variation in polyclonal and isotype antibody response.

**Fig. 9:**
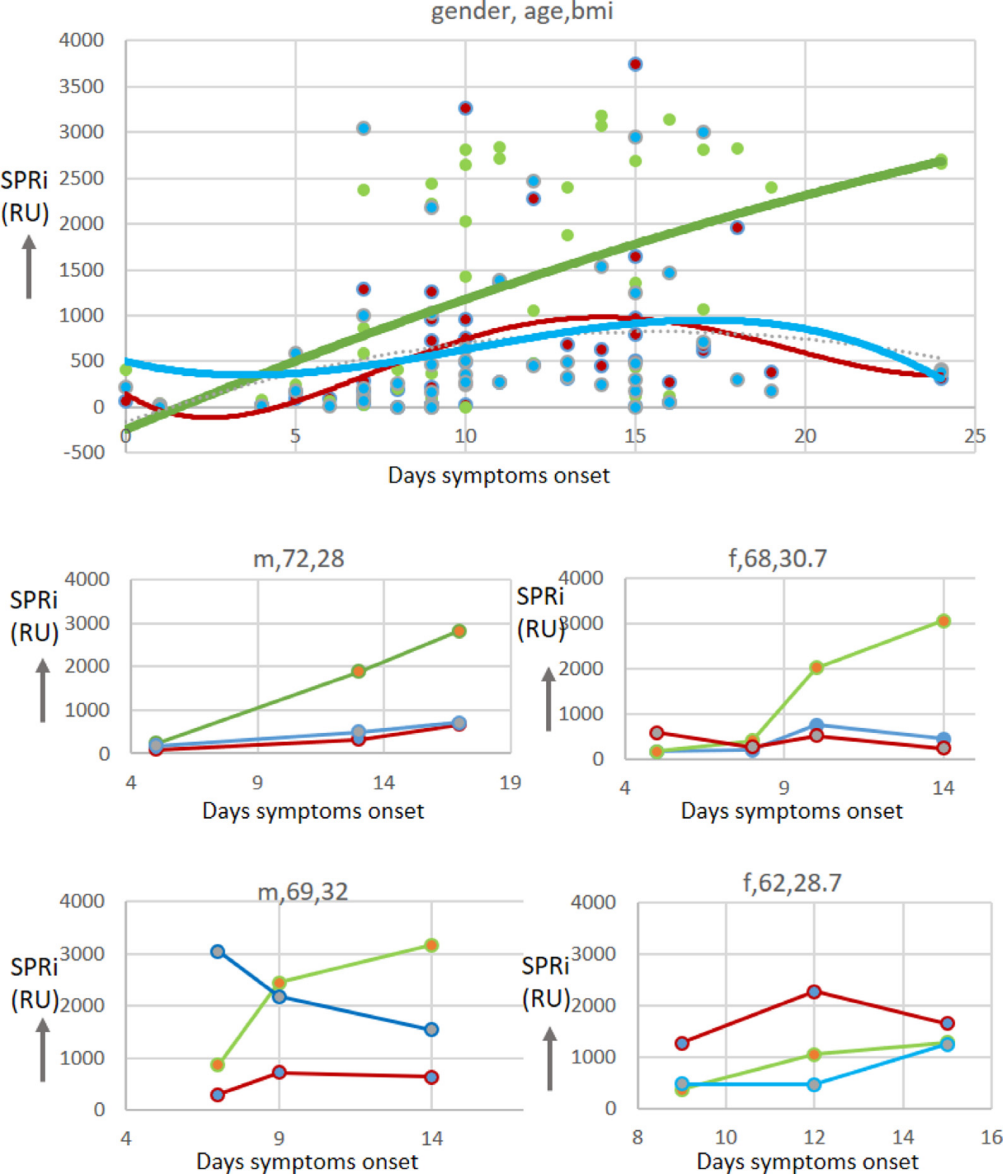
The levels of IgM (magenta), IgG (green) and IgA (azur, blue) as a function of days of symptoms onset for a few patients. The first top panel shows the values of all the tested patients characterized by gender, age and bmi. A wide variety of levels of the antibodies was measured and polynomial trend lines are plotted. In the 4 remaining panels typical immunological courses of the disease are shown. We found a direct correlation of severity of the disease with the level of the antibody response. See Fig. 2. of the main article [Schasfoort, R.B.M., et al. Biosensors and Bioelectronics 183 (2021) 113165]

**Fig. 10:**
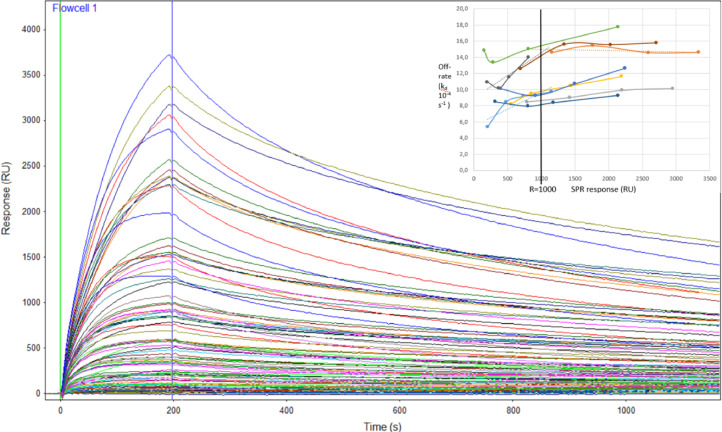
Sensorgram of a Fab anti human IgG injection and the response of all 96 spots. In the dissociation phase of the sensorgram one can observe differences due to stronger or weaker binding of the anti-RBD antibodies to the spots. The off-rate is however also dependent on the ligand density caused by the rebinding effect. Therefore, we decided that the anti-RBD antibodies from the same patient should be spotted in 4 densities. Panel insert: The off-rate as a function of the Fab response. The value of the off-rate was determined at R=1000 RU by interpolating the values obtained in the ligand density gradient according to Schasfoort, R.B.M. et al., 2016.

**Fig. 11:**
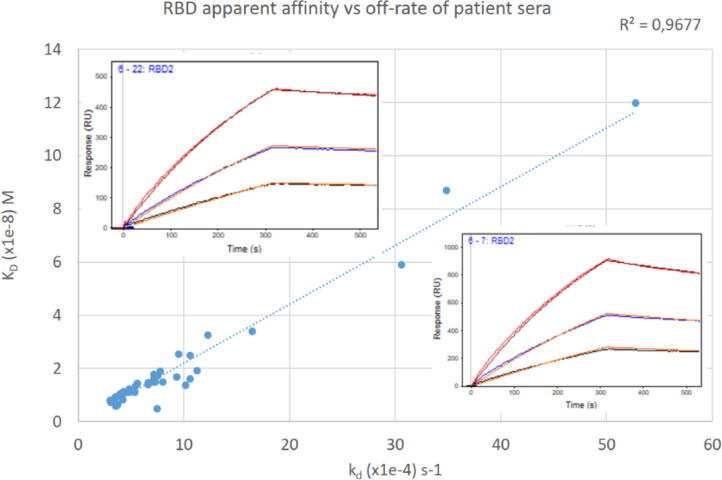
Classical SPR test in low throughput for affinity profiling patient antibody binding. Instead of high throughput spotting, patient sera were injected after each other in 3 serial dilutions in the SPRi imager on an RBD sensor with spots with various ligand density. The association and dissociation rate of the anti-RBD response was calculated in Scrubber (BioLogic Software, Australia) with a 1:1 fitting algorithm. The equilibrium dissociation constant (K_D_) for each serum was plotted against the off-rate (k_d_) and an apparent linear relation between K_D_ and k_d_ was found. Because of this strong correlation of K_D_ and k_d_, we decided for the high-throughput measurements in the LSA and IBIS MX96 to measure only the off-rates (for all 384 patients simultaneously).

The following question remains: Does the off-rate of the specific polyclonal isotype antibodies solely reflects the affinity equilibrium constant and can this parameter be used to reveal the strength of binding of the patients anti-SARS-CoV-2 antibodies? We checked this with a classical SPR test in low throughput for affinity profiling of patient antibody binding. Instead of high throughput spotting, individual patient sera were injected after each other in 3 serial dilutions in the SPRi imager on an RBD sensor over spots with various ligand density. The association and dissociation rate of the anti-RBD response was calculated in Scrubber (BioLogic Software, Australia) with a 1:1 fitting algorithm. The equilibrium dissociation constant (K_D_) for each serum was plotted against the off-rate (k_d_) and an apparent linear relation between KD and kd was found (see Fig. 11). Because of this strong correlation of K_D_ and k_d_, we decided for the high-throughput measurements in the LSA and IBIS MX96 to measure only the off-rates (for all 384 patients simultaneously).

In a single automated experiment, the strength of binding can be measured plus the concentration of IgM, IgG and IgA in the serum of CoViD-19 patients and this can be considered as the first break-through of the high-throughput method. The second break-through is that we observed that during the course of the disease the strength of binding of the anti RBD antibodies of CoViD-19 patients increases independently from the isotype ratios which changed too. Hence, a well-known maturation effect of the affinity of antibodies can be ranked and quantified precisely. It is clear that the new method enables to follow the strength of binding and concentration of anti-RBD antibodies not only of CoViD-19 patients but also of healthy people who will be vaccinated to SARS-CoV-2 in large clinical vaccination programs that are foreseen for 2021 .

## Author contributions

RS performed the SPRi study, analyzed the data and wrote the manuscript with input from all other authors. RS, MKa, HK, LM and LT designed the study. JvW contributed to the IgA implementation and discussions with RS. MvA wrote the biphasic software program that was essential for calculating the antibody R_max_ values. JP performed the data analysis and measurements on the LSA equipment of Carterra. JH and MKo contributed to details of spotting and buffer compositions in the SPRi test. MKa helped to organize the manuscript and edited it extensively. AM performed the MGA-experiments with the patients in the hospital. MB supported with additional information and changed the focus of the TFF project to enable this study. HK supported with patient data collected at the hospital. LT supervised and edited extensively and the clinical diagnostic study was performed under supervision of LM.

## References

[1] Alhazzani W. (2020). Surviving Sepsis Campaign Guidelines on the Management of Adults With Coronavirus Disease 2019 (COVID-19) in the ICU: First Update. Crit Care Med.

[2] Lan J. (2020). Structure of the SARS-CoV-2 spike receptor-binding domain bound to the ACE2 receptor. Nature.

[3] Meyer B. (2014). Serological assays for emerging coronaviruses: challenges and pitfalls. Virus research.

[4] Schasfoort R.B.M. (2016). Interpolation method for accurate affinity ranking of arrayed ligand–analyte interactions. Anal. Biochem..

[5] Schasfoort R.B.M. (2021). Presence and strength of binding of IgM, IgG and IgA antibodies against SARS-CoV-2 during CoViD-19 infection. Biosens. Bioelectron..

[6] Zhu X. (2013). Receptor-binding domain as a target for developing SARS vaccines. J Thorac Dis.

